# Genetic Characterization of HIV-1 Subtype D Near-Full-Length Proviral Genomes by Illumina Massively Parallel Sequencing Technology

**DOI:** 10.1128/genomeA.00586-14

**Published:** 2014-06-12

**Authors:** Rodrigo Pessôa, Maria Esther Lopes, Sabri S. Sanabani

**Affiliations:** a Department of Virology, São Paulo Institute of Tropical Medicine, University of São Paulo, São Paulo, Brazil; b Hemorio, Rio de Janeiro, Brazil; c Department of Pathology, LIM 03, Hospital das Clínicas (HC), School of Medicine, University of São Paulo, São Paulo, Brazil

## Abstract

This study describes the near-full-length genome deep sequencing of two HIV-1 subtype D strains identified in blood donors in Rio de Janeiro, Brazil, in what seems to have been a small restricted subtype D epidemic in the country.

## GENOME ANNOUNCEMENT

Brazil, the world's fifth most populous country after China, accounts for about one-third of the HIV infections in Latin America, with an estimated 718,000 people infected (http://www.aids.gov.br). Most of these infections are caused by HIV-1 subtype B, except in the southern region, where subtype C prevails (1). HIV-1 subtype D viruses were first isolated from the peripheral blood lymphocytes in 1983 in patients from the Democratic Republic of the Congo (DRC) (2). Subtype D was also reported from Brazil in a dually infected individual in 1996 (3). Since then, there have been only sporadic cases with subtype D infection detected in the Rio de Janeiro (RJ) state (southeastern region) (4–7), but none were comprehensively sequenced. In this study, we report the first deep proviral genome sequencing of two HIV-1 subtype D variants obtained between 2007 and 2011 from Retrovirus Epidemiology Donor Study-II (REDS-II) blood donors in RJ.

Cellular DNA was extracted from 5 peripheral blood mononuclear cells (PBMC) using the QIAamp blood kit (Qiagen), according to the manufacturer’s instructions. The near-full-length genomes (NFLGs) from five overlapping fragments were obtained by PCR and determined by a previously reported method (8). A sequencing library was prepared as described previously (9). Briefly, the amplified fragments from a single viral genome were purified, quantified, and pooled together at equimolar ratios. Approximately 1 ng of each pool was used in a fragmentation reaction. Finally, all libraries were pooled and loaded onto an Illumina MiSeq for paired-end 250-bp sequencing. Fastq files were generated, validated, and *de novo* assembled into contiguous sequences and annotated with CLC Genomics Workbench version 5.5. Maximum likelihood trees were obtained by PhyML version 3.1 using the GTR+I+G model (10). The approximate likelihood ratio test was used as a statistical test to calculate branch support.

The ultradeep sequencing yielded >1.6 × 10^6^ sequences reads, with average coverages ranging from 254× (10BR_RJ095) to 2,372× (10BR_RJ108). To determine the phylogenetic relationships of the newly characterized viruses, we constructed evolutionary trees from the NFLG consensus sequences. The results confirmed the initial diversity observed among subtype D previously described in the *pol* gene of HIV-1 (11). The intrasubtype distance for the two Brazilian variants was 8.1% and was comparable to the distances observed between subtypes D from different geographic locales. The close relationship of these Brazilian subtype D variants with sequences from Tanzania confirms an African origin for the subtype D circulation in Brazil. As inferred by geno2pheno coreceptor (12), both sequences were predicted to be X4 viruses.

This study describes the first NFLG HIV-1 subtype D viruses from South America. Despite early detection of subtype D in Brazil, it seemed not to have spread in much the same epidemic proportions as did subtype B or BF1 infections, which might imply that it was introduced and contained in only small networks.

### Nucleotide sequence accession numbers.

All consensus genome assemblies generated in this study were submitted to NCBI’s GenBank database under accession no. KJ787683 and KJ787684.
